# Proteomics-Based Retinal Target Engagement Analysis and Retina-Targeted Delivery of 17β-Estradiol by the DHED Prodrug for Ocular Neurotherapy in Males

**DOI:** 10.3390/pharmaceutics13091392

**Published:** 2021-09-02

**Authors:** Katalin Prokai-Tatrai, Khadiza Zaman, Vien Nguyen, Daniel L. De La Cruz, Laszlo Prokai

**Affiliations:** Department of Pharmacology and Neuroscience, University of North Texas Health Science Center, Fort Worth, TX 76107, USA; khadiza.zaman@unthsc.edu (K.Z.); Vien.Nguyen@unthsc.edu (V.N.); DanielDeLaCruz@my.unthsc.edu (D.L.D.L.C.); Laszlo.Prokai@unthsc.edu (L.P.)

**Keywords:** DHED prodrug, 17β-estradiol, E2, eye drops, glaucoma, male retina, parallel-reaction monitoring, proteomics, neuroprotection, LC-MS/MS, isotope-dilution, targeted proteomics

## Abstract

We examined the impact of 17β-estradiol (E2) eye drops on the modulation of the proteome profile in the male rat retina. With discovery-driven proteomics, we have identified proteins that were regulated by our treatment. These proteins were assembled to several bioinformatics-based networks implicating E2’s beneficial effects on the male rat retina in a broad context of ocular neuroprotection including the maintenance of retinal homeostasis, facilitation of efficient disposal of damaged proteins, and mitochondrial respiratory chain biogenesis. We have also shown for the first time that the hormone’s beneficial effects on the male retina can be constrained to this target site by treatment with the bioprecursor prodrug, DHED. A large concentration of E2 was produced after DHED eye drops not only in male rat retinae but also in those of rabbits. However, DHED treatment did not increase circulating E2 levels, thereby ensuring therapeutic safety in males. Targeted proteomics focusing on selected biomarkers of E2’s target engagement further confirmed the prodrug’s metabolism to E2 in the male retina and indicated that the retinal impact of DHED treatment was identical to that of the direct E2 treatment. Altogether, our study shows the potential of topical DHED therapy for an efficacious and safe protection of the male retina without the unwanted hormonal side-effects associated with current estrogen therapies.

## 1. Introduction

Accumulating evidence shows that 17β-estradiol (E2) impacts the retina and, in general, the central nervous system (CNS) through well-orchestrated genomic and nongenomic actions that tackle numerous pathways implicated in CNS-related pathological processes [1,2]. These processes include the initiation and propagation of neurodegeneration currently without viable therapeutic options [3,4,5]. One of the most common ocular neurodegenerations is seen in glaucoma [6]. In this group of diseases, gradual and irreversible damage to the retinal ganglion cells (RGCs) and their axons lead to visual disability or even blindness in spite of controlling the intraocular pressure (IOP) [7,8]. Protection of RGCs is imperative, as they are the only neurons in the retina whose axons form the optic nerve that carries visual information to the retinorecipient areas in the brain [9].

We have previously reported the beneficial effect of topical E2 on retinal health in an animal model of human glaucoma using female Brown-Norway rats [10,11]. We have shown that E2 eye drops provided therapeutic drug concentration in the retina that prevented visual function impairment in the hypertensive female rat eye after episcleral vein injection of hyperosmotic saline [12]. In a follow-up study, we have also shown the positive impact of E2 eye drops on the protein networks in the context of biological processes in the female rat retina through quantitative proteomics [13]. We have presented several protein networks with associated canonical pathways that evidenced retinal target engagement of E2 as a broad-spectrum neuroprotectant. Altogether, these findings provide independent confirmations on the therapeutic benefits of E2 for the female retinal health [14,15]. In addition, they complement previous reports on the detrimental effect of estrogen deficiency on visual function in females not only through animal studies [16,17] but also epidemiological observations [18]. Postmenopausal women face a higher risk of developing glaucoma than prior to becoming estrogen-deficient [19]. Moreover, it has been shown that an early loss of endogenous E2 in younger women results in premature aging and excessive vulnerability of the optic disc to glaucomatous neurodegeneration [20].

**Figure 1:**
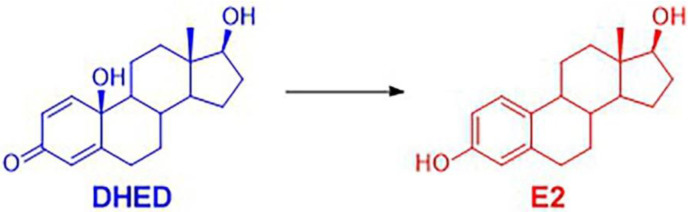
The topically applied 10β,17β-dihydroxyestra-1,4-dien-3-one (DHED) bioprecursor prodrug metabolizes to the neuroprotective 17β-estradiol (E2) in the male retina without exposing the circulation to E2.

Even though women are more often affected, glaucoma and several other ocular neurodegenerative diseases are age-related and also affect men [21,22,23]. E2 formed from testosterone by aromatase and its nuclear receptors are also found in the male CNS, including the retina [24,25]. Accordingly, it is reasonable to assume that estrogen receptors also play an important role in the pathophysiology of ocular neurodegeneration and its treatment in males. On the other hand, retinal neuroprotection in males cannot be addressed with direct administration of E2 because long-term therapy is needed to control ocular neurodegeneration. This, in turn, inevitably exposes the entire body to unwanted side effects of the hormone, including feminization. Therefore, retina-targeted E2 delivery is especially important in males to ensure therapeutic safety [26]. We have previously utilized 10β,17β-dihydroxyestra-1,4-dien-3-one (DHED) eye drops for successful retina-targeted E2 delivery in female animals [11]. This topically administered DHED bioprecursor prodrug metabolized in the female retina to E2 (Figure 1), without producing an elevation in circulating E2 level. It has, however, not been shown before whether this site-specific DHED-to-E2 biotransformation seen in Figure 1 also occurs in the male retina, as gender appears to be a potent modifier in several aspects of the CNS [27,28,29]. 

In the current study, we first established that topically administered E2 exhibited neuroprotective target engagements in the male retina by label-free shotgun proteomics relying on bioinformatics. At the same time, this non-invasive treatment also produced a significant unwanted peripheral exposure to the hormone, making the administration of E2 eye drops unsafe for treating the male retina, especially upon chronic administration, which is needed in ocular neurotherapy. Therefore, next, we investigated for the very first time the viability of our DHED strategy for retina-targeted E2 delivery in males to explore therapeutic safety and efficacy. Additionally, the impact of DHED-derived E2 in the male retina was unequivocally confirmed by targeted proteomics focusing on representative proteins identified by shotgun proteomics in the context of neuroprotection upon topical administration of E2.

## 2. Materials and Methods

### 2.1. Chemicals and Reagents

E2, 2-hydroxypropyl-β-cyclodextrin (HPβCD), urea, dithiothreitol, iodoacetamide, ammonium bicarbonate, and formic acid (ACS reagent grade, ≥98%) were purchased from Millipore Sigma (St. Louis, MO, USA). Furthermore, 10β,17β-dihydroxyestra-1,4-dien-3-one (DHED), 10β,17β-dihydroxyestra-1,4-dien-3-one-16,16,17-d3 (d_3_-DHED), and 10β,17β-dihydroxy-estra-1,4-dien-3-one-2,4,16,16,17-d5 (d_5_-DHED) were synthesized in our laboratory, as reported before [30,31]. Additionally, 17β-Estradiol-13,14,15,16,17,18-^13^C6 (^13^C_6_-E2) with 99% isotope purity was obtained from Cambridge Isotope Laboratories (Andover, MA, USA). Sequencing-grade trypsin was purchased from Promega (Madison, WI, USA). Water and acetonitrile were Optima^®^ LC/MS grade, and supplied by Thermo Fisher Scientific (Waltham, MA, USA). Ketamine (100 mg/mL), Xylazine (20 mg/mL), and SomnaSol (pentobarbital sodium 390 mg/mL) were supplied by Covetrus (Fort Worth, TX, USA). Stable-isotope labeled proteotypic peptides as internal standards (SISs) were obtained from Vivitide, LLC (Gartner, MA, USA).

### 2.2. Animals and Treatments

All procedures conformed to the ARVO Statement for the Use of Animals in Ophthalmic and Vision Research. Before the initiation of the studies, all protocols were approved by the Institutional Animal Care and Use Committee at the University of North Texas Health Science Center (approval numbers: 2019-0033 approved on 11 July 2019 and 2019-0016 approved on 4 June 2019). Orchiectomized (ORX) Brown Norway rats weighing 200–250 g were purchased from Charles Rivers Laboratories (Wilmington, DE, USA). Male New Zealand white rabbits weighing 1.0–1.5 kg were also ordered from Charles Rivers Laboratories. Ten rats received 10 µL of E2 or DHED eye drop once daily for three weeks [11,13]. The filtered eye drops contained 0.1% (*w/v*) test agent in saline vehicle containing 20% (*w/v*) HBβCD. Ten control rats received 10 µL of this vehicle as eye drops for the same dosing regimen and duration. Animals were euthanized 24 h after the last treatment. From the anesthetized animals (i.p. administration of 60 mg/kg ketamine and 10 mg/kg xylazine) blood was collected by cardiac puncture in a BD Vacutainer (Fisher Sci., Atlanta, GA, USA) to make serum. Animals were then perfused with 3 × 10 mL ice-cold saline through the left ventricle to eliminate blood from tissues to avoid bias associated with drug quantitation. The eyes were enucleated immediately followed by quick dissection to collect the retina and major eye parts. Other off-target body parts, including the seminal vesicle (SV) and anterior pituitary (AP), were also harvested from each animal to record their wet weight. 

### 2.3. Sample Preparations for Proteomics and Drug Quantitation Studies

For proteomics studies, the retinae were rinsed with saline and then blotted dry. For drug quantitation for E2 and DHED, or for their stable-isotope-labeled isomers when applicable, retina homogenates (in 400 µL of pH 7.4 phosphate buffer) were prepared, spiked with 100 pg of ^13^C_6_-E2 and 1000 pg of d_5_-DHED as internal standards (ISs), and extracted with four volumes of tert-butyl methyl ether. The organic layers obtained from the liquid–liquid extractions were removed and aliquoted. One third of the organic layer was transferred to a reacti-vial (Supelco, Bellefonte, PA, USA) and evaporated under a nitrogen stream to yield samples for derivatization with dansyl chloride (Dns-Cl) and subsequent liquid chromatography–tandem mass spectrometry (LC-MS/MS) analysis for E2 (or for d_3_-E2, when applicable) [32]. The dansylated samples were centrifuged, transferred to autosampler vials, sealed, and assayed. The other portion of the extract was evaporated separately for DHED quantitation [11,30]. Sera (100 μL) were extracted and processed analogously. In the drug distribution study, rats were euthanized at predetermined time points (0.25, 0.5, 1, 2, 4, 6, 12, and 24 h) after administration of a single eye drop of DHED to *n* = 4 animals per time point. Tissue processing was the same as for the 3-week treatment. Rabbits received a single 40 µL of eye drop of d_3_-DHED to avoid potential bias associated with circulating endogenous E2. These animals were anesthetized with intramuscular (i.m.) ketamine (100 mg/kg) and xylazine (10 mg /kg) followed by injection of SomnaSol to the heart at a dose of 100 mg/kg body weight. Tissues were collected and processed as for rats for subsequent drug quantitation. Simultaneous measurements of d_3_-E2 and endogenous E2 were done using ^13^C_6_-E2 [33] as the IS for the isomeric estrogens, and d_5_-DHED was used as the IS for d_3_-DHED quantitation [11,30]. 

### 2.4. Global Label-Free Proteomics and Bioinformatics

#### 2.4.1. Sample Preparation

To dissolve proteins of the retinae, each isolated retina was incubated in 200 µL of 8 M aqueous urea solution for 60 min, and subsequently centrifuged for 5 min at 1400 g, as reported before [13,34]. After collecting the supernatant, protein contents were estimated using a Microplate reader (BioTek Synergy H1 with Take3 plates, Agilent, Palo Alto, CA, USA), and the samples were aliquoted to contain 100 µg protein each. The volume of the aliquoted samples was adjusted to 100 µL with 25 mm NH_4_HCO_3_ solution followed by reduction with dithiothreitol (1 mM at 65 °C for 30 min) and carbamidomethylation (5 mM iodoacetamide at room temperature and in the dark for 30 min). After 9-fold dilution of the reduced and alkylated sample with aqueous 25 mM ammonium bicarbonate solution, sequencing-grade trypsin (2 µg) was added to digest the proteins overnight at 37 °C, after which the digestion was quenched with 5 µL of formic acid. The samples were desalted by solid-phase extraction using 1-mL Sep-Pak™ C-18 cartridges (Waters USA, Milford, MA), and then the extracts were dried under vacuum (Vacufuge™, Eppendorf AG, Hamburg, Germany) into 1.5-mL centrifuge tubes. The dried residues were dissolved in an aqueous medium containing 5% (*v/v*) and 0.1% (*v/v*) formic acid to produce 1 µg/µL protein content.

#### 2.4.2. LC–MS/MS Analyses

Data-dependent LC–ESI-MS/MS (ESI denotes electrospray ionization) acquisitions were used, as described previously [13,34]. Briefly, the system consisted of a Thermo Scientific (San Jose, CA, USA) LTQ Orbitrap Velos Pro mass spectrometer connected to EASY nLC-1000 nanoflow LC, and attached to an Easy nanospray system. Separations were done using 15 cm × 75 μm i.d. Pepmap^TM^ RSLC C18 column packed with 3-µm C18 particles (Thermo Fisher Scientific, San Jose, CA, USA). Gradient elution was performed using water that contained 0.1% (*v/v*) formic acid as solvent A and acetonitrile containing 0.1% (*v/v*) formic acid as solvent B after the injection of 5 µL sample solution and 20 min column equilibration at 5% B while maintaining a constant column pressure at 600 bar. The peptides were eluted at 300 nL/min flow rate using the following gradient program: (i) isocratic at 5% B for 5 min; (ii) linear gradient to 40% B over 90 min and then (iii) 5 min of isocratic at 40% B; (iv) scaled up to 90% B for 5 min; (v) isocratic flow at 90% B for 5 min; and (vi) returning back to 5% B in 20 min. The ion source of the mass spectrometer was operated in positive-ion nanoESI mode with a source voltage of 2.0 kV and an ion-transfer tube temperature of 275 °C. Full-scan mass spectra were acquired at nominal mass resolution of 60,000 (at *m/z* 400) in the Orbitrap, and up to 20 data-dependent tandem mass spectra (MS/MS) were obtained in the LTQ Velos ion trap. Each full MS/MS scan was acquired using collision-induced dissociation (CID, at 35% normalized collision energy) of multiply charged ions (z ≥ 2). After a precursor ion was selected for fragmentation, it was dynamically excluded from MS/MS analyses for 60 s.

#### 2.4.3. Processing of Raw Data and Label-Free Quantification

MS/MS spectra were searched against the UniProt protein sequence database (species: *Rattus norvegicus*, 2019; 29938 entries), using the Mascot search algorithm (version 2.6.2; Matrix Science, Boston, MA, USA) run within the Proteome Discoverer software (version 2.3; Thermo Fisher Scientific) [13]. Briefly, parent ion tolerance and fragment ion mass tolerance were set to 25 ppm and 0.80 Da, respectively, and one missed cleavage was set as a search filter. Fixed and variable modifications included carbamidomethylation of cysteine and oxidation of methionine, respectively. Search results were validated to meet strong criteria of protein identifications using the Peptide Prophet [35] and Protein Prophet [36] algorithms requiring >95% and >99% probabilities, respectively, and at least two identified unique peptides for each protein, using the Scaffold software (version 4.9.0, Proteome Software Inc.; Portland, OR, USA). Label-free quantification (LFQ) relied on spectral counting (SC) [34] built into the Scaffold software, and *p* < 0.05 was considered significantly different using unpaired *t*-tests for statistical comparison of protein expression levels between sample categories. Missing values, if there were any, were handled using Scaffold’s default method and settings.

#### 2.4.4. Bioinformatics

The identified E2-regulated proteins were submitted to Ingenuity Pathway Analysis^®^ (IPA^®^, QIAGEN, Redwood City, CA, USA) for annotations and building protein interaction networks, along with the identification of associated biological functions and processes. Overlaps of *p*-values relied on IPA^®^’s calculations using the right-tailed Fisher’s exact test [37].

### 2.5. PRM-Based Targeted Proteomics by LC–MS/MS and Data Analysis

For targeted PRM method development, we selected biologically significant proteins involved in the context of ocular neuropathies from our global discovery-driven analysis. The approach relied on the use of SIS peptides added to the tissue extracts for comparison against native tryptic peptide (NAT) levels in the digested samples. The sequence, spectrum, and fragmentation table of proteotryptic target peptides from the corresponding proteins were obtained from our data-dependent label-free shotgun analysis using the Scaffold software. It was ensured that the selected peptides did not have any missed cleavages or any extensive post translational modifications. Peptides were filtered based on 100% peptide identification probability, peptide charge (z) of 2, and retention time filtering as shown in Table S3. SIS peptides (Table S3) were spiked in the sample before reduction, alkylation, and digestion. The chromatographic and nanoESI conditions were similar to those described for the discovery-driven method (Section 2.4.2). Full MS/MS scans were acquired using a 2 (*m/z*) isolation width, CID at 35% normalized collision energy and a 30 ms activation time. The Orbitrap’s mass resolution was set to 15,000 (at *m/z* 400).

Scaffold peptide ID file extensions with mzid extensions were exported from Scaffold and used to generate peptide spectral libraries in the publicly available Skyline software (v.1.21.1.0.146) [38], with trypsin as digesting enzyme, one missed cleavage, and modifications set to carbamidomethylation of cysteine and oxidation of methionine. Sequences of target proteins and shotgun proteomics were obtained by creating various spectral libraries with our own shotgun proteomics study performed using the LTQ Orbitrap Velos Pro (see details in Section 2.4.2 above) and publicly available databases from the National Institute of Standards and Technology [39], and imported into Skyline, populating a spectral tree. Retention time scheduling was performed by importing raw data files of target SIS peptides. Experimental raw data files were imported, and precursor and product ion chromatograms were extracted and analyzed under the PRM mode. For selecting MS/MS transitions, the precursor charges were set to 2 product ion charges, 1 and 2, with b and y fragment ions considered. Acquisition method, product-mass analyzer, and resolving power were set to targeted, Orbitrap, and 15,000, respectively. Ion match tolerance was 0.5 Da to search for product ions. Transitions reported by Skyline were used for relative quantitation by first taking the average from the technical replicates of all samples for each peptide (Table S4) and then using the NAT peptide to SIS peptide ratios for obtaining fold changes in treated samples compared to controls. For validation, the peptide sequence was confirmed by the acquired MS/MS scan from b and y sequence ions marked according to the nomenclature by Roepstorff and Fohlman [40]. 

### 2.6. LC-MS Analysis for Prodrug Quantitation

DHED and d_3_-DHED quantitation was based on the principles of isotope dilution using validated assays [33]. Samples were analyzed on a TSQ Quantum Ultra triple-quadrupole mass spectrometer (TSQ, Thermo Electron Corporation, Trace Chemical Analysis, Austin, TX, USA) using positive-ion atmospheric pressure chemical ionization (APCI). The instrument was operated with Xcalibur (version 2.2., Thermo Fisher Scientific, Waltham, MA, USA) data acquisition software. Gradient separations were carried out using a Vanquish ultra-high performance liquid chromatography (UHPLC) system (Thermo Electron Corporation). For APCI analysis of DHED or d_3_-DHED [11], LC separation was carried out on a Supelco (Bellefonte, PA, USA) Discovery HS C-18 reversed-phase column (50 × 2.1 mm, 5 μm), using isocratic elution with a flow rate of 0.3 mL/min. The eluent system consisted of (A) 1% (*v/v*) acetic acid in water and (B) 1% (*v/v*) acetic acid in acetonitrile. Isocratic eluent composition was set at 35% (*v/v*) solvent B. SRM transitions of *m/z* 289 → 123, 292 → 123, 294 → 125 were used for DHED, d_3_-DHED, and d_5_-DHED, respectively. 

### 2.7. LC-MS Analysis for Estrogen Quantitation

E2 and d3-E2 quantitation after eye drop treatments [11,30] were analyzed on the same MS instrument as described under Section 2.6, but in a positive-ion heated ESI (HESI) mode. A Kinetex phenyl-hexyl UPLC column (100 Å, 50 × 2.1 mm, 1.7 µm) from Phenomenex (Torrance, CA, USA) was used for chromatographic separations at 0.4 mL/min flow rate. SRM was set up to *m/z* 506 → 171, 509 → 171, and 512 → 171 for Dns-E2, Dns-d_3_-E2, and Dns-^13^C_6_-E2, respectively. 

### 2.8. Statistical Analysis 

Descriptive statistics were calculated for each group and for all outcomes. Differences of mean values between experimental groups were assessed by one-way ANOVA followed by post hoc Tukey tests. In all statistical analyses, *p* < 0.05 was considered statistically significant. 

## 3. Results 

### 3.1. Label-Free Shotgun Proteomics Revealed E2’s Target Engagements in the Male Rat Retina upon Bioinformatic Analyses 

In this study, ORX male Brown Norway rats with no endogenous E2 source received 0.1% (*w/v*) E2 eye drops once daily (q.d.) for 3 weeks, similar to an earlier study using ovariectomized female animals [10,13]. Retinae were collected 24 h after the last treatment. Our shotgun proteomics relying on data-dependent nanoflow LC–MS/MS and LFQ covered 1761 proteins with 0.02% and 0.6% false discovery at the peptide and protein levels, respectively, according to a decoy-based method of estimation (Table S1). Of the 1761 confidently identified proteins using stringent criteria described in Section 2.4.3 and SC as the method of LFQ [34], 130 proteins were up-regulated, while 9 proteins were down-regulated significantly by topically administered E2 in the retina of these rats (Table S2).

**Figure 2:**
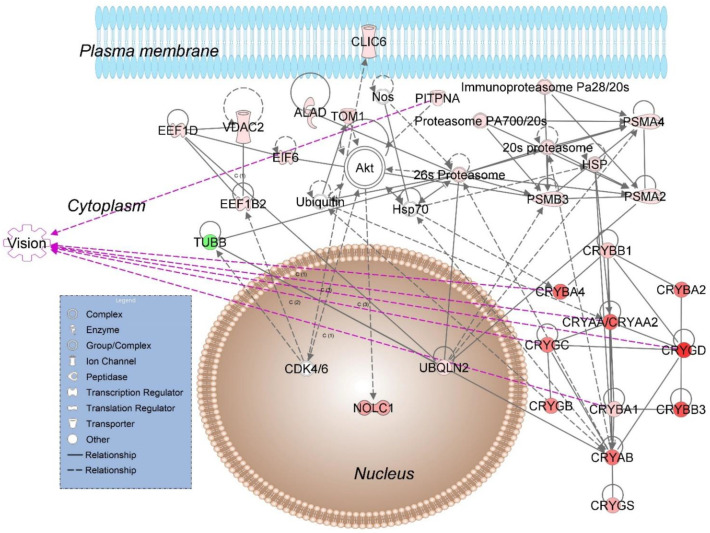
A representative IPA^®^ network assembled from E2-regulated proteins in the retina of Brown Norway male rats associated with development disorder, ophthalmic disease, as well as organismal injury and abnormalities. Abbreviations: 20 s proteasome; 26 s proteasome; ALAD, aminolevulinate dehydratase; CDK4/6, cyclin D1 group; CLIC6, chloride intracellular channel 6; CRYAA, CRYAA2, CRYAB, CRYBA1, CRYBA2, CRYBA4, CRYBB1, CRYBB3, CRYGB, CRYGC, CRYGD, CRYGS, crystallines; EE1FB2, elongation factor 1-beta; EEF1D, elongation factor 1-delta; E1F6, eukaryotic translation initiation factor 6; HSP, heat shock protein; HSP70, heat shock protein 70; NOLC1, nucleolar and coiled body phosphoprotein 1; Nos, neuronal nitric oxide synthase; PITPNA, phosphatidylinositol transfer protein α; proteasome PA700/20s; immunoproteasome Pa28/20s; PSMA2, 20S proteasome α2-subunit; PSMA4, 20S proteasome α4-subunit; PSMB3, proteasome (prosome, macropain) β-subunit, type 3; TOM1, target of myb1 membrane trafficking protein; TUBB, β-tubulin 1; UBQLN2, ubiquilin 2; and VDAC2, voltage dependent anion channel 2. The shapes (see legend in blue box) represent molecular classes of proteins. Red and green colors denote upregulation and downregulation in response to E_2_ treatment, respectively. The intensity of color indicates the relative magnitude of fold change in protein expression pattern. Purple arrows indicate proteins linked to the top physiological process of visual system development and function (see Table 1B) by the overlay function of IPA^®^.

**Figure 3:**
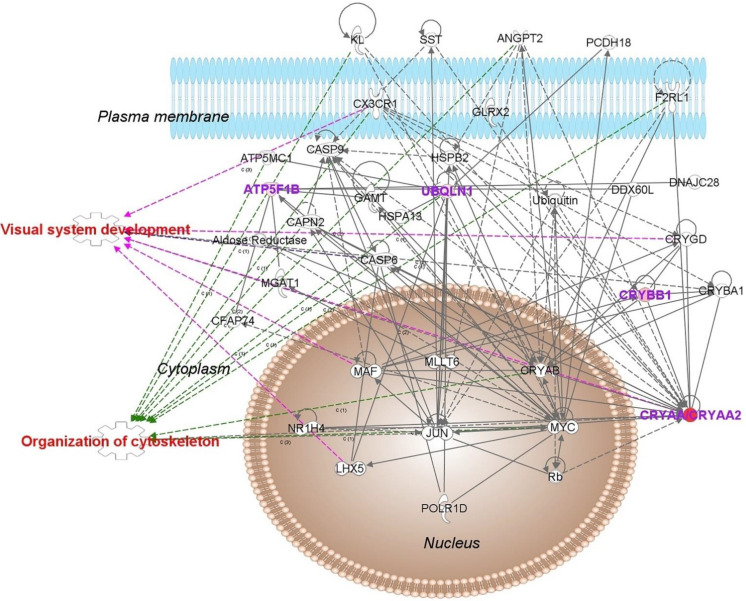
IPA^®^ network assembled from the chosen E2-regulated proteins CRYAA/CRYAA2, CRYBB1, UBQLN1, and ATP5F1B for targeted proteomics in the retina of ORX Brown Norway rats and associated with ophthalmic disease, development disorder, and hereditary disorder. Abbreviations: ANGPT2, angiotensin 2; ATP5MC1, ATP synthase subunit C1; CAPN2, calpain 2; CASP6, caspase 6; CAS9, caspase 9; CFAP74, cilia and flagella associated protein 74; CRYAB, CRYBA1, CRYGD, crystallins; CX3CR1, chemokine receptor; DDX60L,DExD/H-box 60 like; DNAJC28, DnaJ heat shock protein; F2RL1, coagulation factor 2 receptor like 1; GAMT, guadinoacetate methyltransferase; GLRX2, glutaredoxin 2; HSPA13, heat shock protein 70 family; HSPB2, heat shock protein 2; JUN, Jun protooncogene; KL, alpha KLOTHO; LHX5, LIM homeobox 5; MAF, MAF bZIP transcription factor; MGAT1, mannoside acetylglucosaminyltransferase; MLLT6, myeloid/lymphoid mixed lineage leukemia protein; MYC, MYC protooncogene; NR1H4, nuclear receptor 1 subfamily H; PCDH18, protocadherin 18; POLR1D, polymerase 1 polypeptide D; Rb, Rb tumor suppressor protein; SST, somatostatin. Purple arrows indicate proteins linked to the top physiological process of visual system development and function, and green arrows indicate proteins linked to organization of cytoskeleton-related function (see Table 3) constructed by the overlay function of IPA^®^.

When analyzed by bioinformatics (IPA^®^), E2-regulated retinal proteins in the male rat retina revealed top molecular and cellular functions, as well as physiological development and function summarized in Table 1. Using IPA^®^ we assembled 9 interaction networks from these proteins. A representative network linked to development disorder, ophthalmic disease, as well as organismal injury and abnormalities is shown in Figure 2. Its top canonical pathways included protein ubiquitination and synaptogenesis signaling, as summarized in Table 2. The presence of crystallins strongly associated with vision [41] was also noteworthy. Overall, all the isoforms of these proteins covered by our shotgun proteomics were significantly upregulated based on LFQ in the male rat retina following topical E2 treatments (Table S2). Details on the remaining networks are included in the Supplementary Material (Figures S1–S9). We chose a subset of E2-regulated retinal proteins mapped by IPA^®^ (Figure 2 and Figures S1–S9) with relevance to neuroprotection, and developed targeted proteomic assays relying on the use of stable-isotope labeled proteotypic peptides as internal standards, LC–MS/MS, and parallel reaction monitoring (PRM) [42] to survey crucial biomarkers indicating therapeutic target engagements. As summarized in Figure 3 and Table 3, these proteins assembled an IPA^®^ network associated with visual system development and organization of cytoskeleton. Additionally, this network captured E2’s beneficial effects on the male rat retina in a broad context of ophthalmic disease, development disorder, and hereditary disorder with the top canonical pathways of protein ubiquitination, γ-aminobutyric acid (GABA) receptor signaling, and mitochondrial dysfunction. Upregulation of adenosine triphosphate (ATP) synthase, as a representative estrogen-regulated protein, indicated a favorable impact of the hormone by mediating mitochondrial respiratory chain biogenesis [43]. 

### 3.2. DHED Eye Drops Deliver E2 into the Male Retina without Exposing the Periphery to the Hormone

**Figure 4:**
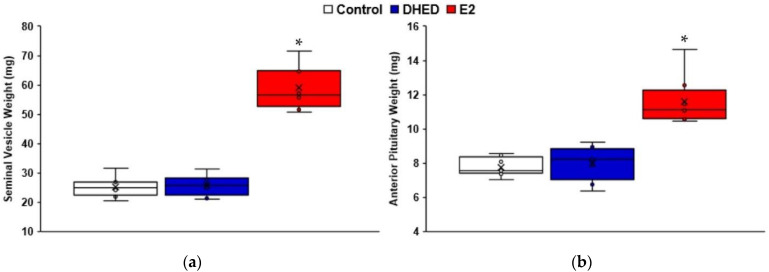
Comparison of wet weights of estrogen–sensitive peripheral organs after three-week q.d. DHED eye drop treatments (10 μL, 0.1% *w/v* in 20% *w/v* HPβCD in saline solution) of ORX Brown-Norway male rats: (**a**) anterior pituitary weights (mg) and (**b**) seminal vesicle weights (mg). Control animals received vehicle eye drops only. Tissue weights were measured 24 h after last treatment. * Statistically significant differences from vehicle and DHED treatments (ANOVA followed by Tukey test, *n* = 10, *p* < 0.05).

Since our global proteomics studies have shown the beneficial impacts of topical E2 treatment on the modulation of retinal proteome profile of ORX rats, next we investigated whether E2’s bioprecursor prodrug (DHED, Figure 1), shown earlier to form E2 in the female retina [11], could also be useful for targeting E2 into the male retina. Therefore, in this part of our study, ORX animals also received q.d. DHED eye drops for three weeks [10,13]. When tissues were collected 24 h after the last eye drop treatment, the retina’s E2 content was 482 ± 77 pg/g (Table 4), prominently indicating that in the male rat retina DHED’s enzyme-catalyzed biotransformation/metabolism to the parent hormone also takes place (Figure 1). This E2 content is comparable to that of direct E2 eye drop treatments (569 ± 169 pg/g, Table 4) used for our proteomics studies. The distinguishing feature of the DHED eye drops is, however, that, unlike direct treatment with topical E2, no increase in circulating E2 level could be established by our validated LC-MS/MS-based bioassay [32]. Accordingly, blood E2 level after 3-week topical DHED treatment was not different from that of the control group receiving the vehicle only, as DHED remained inert in the periphery (Table 4). At the same time, E2 eye drops produced the expected large increase in circulating estrogen (414 ± 79 pg/mL). Concomitantly, as seen in Figure 4a, there was over 40% increase in the AP weight (11.6 ± 1.3 mg) in relation to the control and DHED-treated groups (7.7 ± 0.5 mg and 8.3 ± 1.1 mg), respectively. Alongside, the SV wet weight is practically doubled (59.0 ± 6.8 mg) in the hormone-treated group compared to control (25.2 ± 3.2 mg) or DHED (25.8 ± 2.9 mg) treatment (Figure 4b). These two markers of peripheral estrogen exposure [44,45] are complimentary to the measured significant increase of E2 in the circulation upon topical treatment (Table 4), confirming the unwanted side-effects associated with E2 eye drops in the male animals. 

As tissues were collected 24 h after the last treatment concluding the q.d. 3-week schedule, we used a single DHED eye drop as a reference to survey the retina’s E2 content 24 h post-treatment. Our data show that there was no statistically significant difference between the 3-week and 24-h schedule in this regard; thus, apparently no accumulation of DHED-derived E2 occurred in the male retina over the 3-week treatment schedule. Table 4 also shows that when a stable-isotope-labeled DHED, specifically 10β,17β-dihydroxyestra-1,4-dien-3-one-16,16,17-d_3_ (d_3_-DHED) [26,31] was used, we could not only monitor the endogenous E2 content in the male rat retina, but simultaneously, determine the d_3_-DHED-derived d_3_-E2 at the target site, as well as its absence in the circulation. Table 4 shows that the d_3_-E2 content of the retina (413 ± 130 pg/g) after a single eye drop was comparable with E2 formed from DHED upon identical treatment (397 ± 105 pg/g). 

This measurement also confirmed that DHED indeed metabolizes into E2 in the ORX rat retina, and the significant increase in retinal E2 after DHED eye drops is not due to a DHED-triggered increase in aromatase activity, affecting the endogenous E2 synthesis in the male retina [24]. 

**Figure 5:**
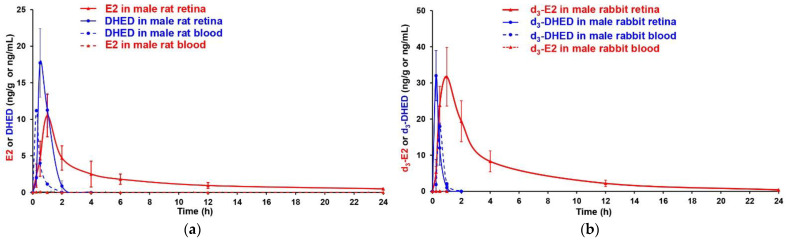
Instillation of (**a**) a single DHED or (**b**) d_3_-DHED eye drop produces the corresponding estrogen in the retina of male rat receiving 10 μL eye drop, as well as in the retina of male New Zealand white rabbit receiving 40 μL of eye drop. The eye drop consisted of 0.1% *w/v* DHED or d_3_-DHED in 20% *w/v* HPβCD in saline. Animals per time point were *n* = 4.

Next, we investigated if topical DHED would also reach and then metabolize within the retina of the male rabbit, having eyes similar in size to those of humans. As the New Zealand white rabbits used for these studies were intact animals, we applied d_3_-DHED eye drops to measure d_3_-E2 in the male rabbit retina. The circulating E2 level in these animals was 10 ± 5 pg/mL. When the rabbits received a single d_3_-DHED eye drop, 24 h post treatment we measured 414 ± 105 pg/g d_3_-E2 in the retina (Figure 4b), while the endogenous E2 in this tissue was 84 ± 19 pg/g. Importantly, no d_3_-E2 was present at this time point in the blood. Figure 5 also shows the time-course of the prodrug’s metabolism to produce the neuroprotective estrogen at the target site, within the retinae of male rats (Figure 5a) and male rabbits (Figure 5b).

There was a similar pattern in terms of the appearance of the hormone after a single prodrug eye drop in the retinae of rats and rabbits. Approximately 60 min after treatment, the maximum E2 content was established in the retina (10.5 ± 2.9 ng/g in rats and 31.7 ± 8.1 ng/g in rabbits), while the prodrug was no longer quantifiable by LC-MS/MS after about 90 min. We also observed the rapid disappearance of DHED (or d_3_-DHED) from the blood in both rats and rabbits. There was a slight transient increase in the circulating E2 around 15-30 min post prodrug treatment (26 ± 10 pg/mL) similar to what we have seen previously in female animals [11,30]. This rapid change quickly disappeared, and the circulating E2 level was no longer distinguishable from that of control, similar to our 3-week treatment schedule (Figure 4 and Table 4). 

Collectively, data shown in Table 4, as well as in Figure 4 and Figure 5, confirm that the convenient and non-invasive topical administration of the DHED prodrug rapidly resulted in the formation of E2 in the male retina without peripheral hormonal liability, which is an especially critical consideration in males to ensure therapeutic safety.

### 3.3. Targeted Proteomics Confirmed E2-Associated Impact in the Male Rat Retina after DHED Eye Drops

**Figure 6:**
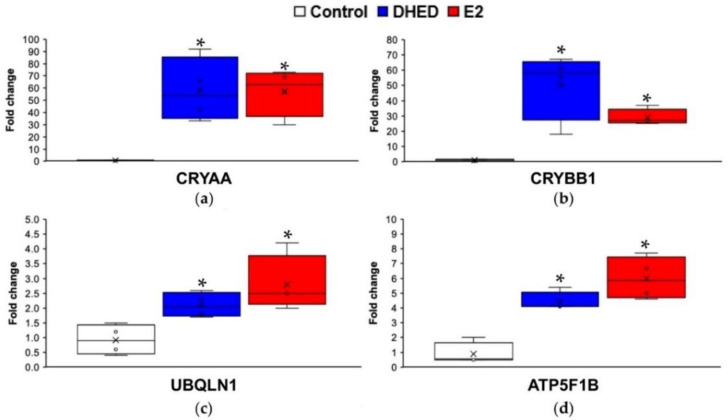
PRM-based targeted proteomics analyzed in Skyline for (**a**) CRYAA, (**b**) CRYBB1, (**c**) UBQLN1, and (**d**) ATP5F1B as representative estrogen-impacted proteins in the retina of ORX Brown Norway rats after eye drop treatments. Both E2 and DHED eye drops showed statistically significant retinal target engagements (indicated by asterisks) compared to control (ANOVA followed by post hoc Tukey test, *p* < 0.05, *n* = 4–5). However, the effects of E2 and DHED treatments on the expression of these marker proteins were statistically indistinguishable.

To further verify that biological impact of treatment with DHED eye drops in the male retina was due to the prodrug’s conversion to E2, we relied on PRM-based targeted proteomics method [42]. We focused on the selected biomarkers (CRYAA, CRYBB, UBQLN1, and ATP5F1B) that captured the hormone’s effects concerning ophthalmic disease, development disorder, and hereditary disorder (Figure 3). Details of the PRM methods for α-crystalline A chain (CRYAA), β-crystalline B chain (CRYBB1), ubiquilin-1 (UBQLN1), and the mitochondrial ATP synthase β-subunit (ATP5F1B) are summarized in Tables S3 and S4. Results seen in Figure 6 indicated that DHED treatment was associated with mechanistic and potential therapeutic target engagements and were congruent with those of direct topical E2 treatment.

## 4. Discussion

Relying on our discovery-driven proteomics approach [13], we have identified several protein networks influenced by E2 in the male rat retina. In the context of potential therapeutic targets for retinal neuroprotection, the top network shown in Figure 2, and linked to development disorder, ophthalmic disease, organismal injury, and abnormalities by IPA^®^, was particularly relevant. This network is dominated by several isoforms of crystallin (CRYAA, CRYAA2, CRYAB, CRYBA1, CRYBA2, CRYBA4, CRYBB1, CRYBB3, CRYGB, CRYGC, CRYGD, and CRYGS). These heterogeneous group of interacting proteins serve diverse functions in the eye [41,46]. In the retina, crystallins maintain homeostasis through their effect on several metabolic and regulatory functions [47], including the regulation of proteasomal activity, as displayed in Figure 2.

Similar to female rats [13], our study has shown that all covered isoforms of crystalline are upregulated in the male rat retina by E2 eye drop administrations (Table S2). Functioning as molecular chaperones that prevent aberrant protein interactions [48,49], α-crystallins protect cells in the retina by inhibiting apoptosis-induced cell death [41,46]. Their abundant presence has been found to promote survival of RGCs upon optic nerve crush and ocular hypertension [50,51], while their suppression has been associated with retinal dystrophy [52] and glaucomatous optic neuropathy [41]. When delivered by intravitreal injection at the time of the IOP increase in a rat model of glaucoma, CRYAB upregulated all subclasses of crystallins in the retina and protected the RGCs from the detrimental effects of increased IOP [53]. Since β-crystallin genes are downregulated at both transcriptional and protein levels in the ocular hypertensive rat retina [53], upregulation of β-crystallins has been implicated in retina neuroprotection and axonal regeneration [54,55]. Like CRYAB, intravitreal injection of CRBB2 has also been shown to improve RGCs’ survival in a rat model of glaucoma by influencing calcium-dependent cell signaling pathways with profound effect on apoptosis and gene regulation [56]. Overall, our data summarized in Figure 2 support that the expression of the interacting group of crystallin genes [57] represents a beneficial target engagement by E2 eliciting neuroprotection of the retina [10].

UBQLN1 was also revealed by bioinformatics as another E2-regulated protein linked to cell death and survival in the male rat retina (Figure S6), but its involvement did not overlap with the IPA^®^ network discussed above. Furthermore, UBQLN1’s network was not only in association with estrogen receptor signaling, but also with a nuclear estrogen receptor (ESR1). Owing to their facilitation of protein disposal by proteasomal and lysosomal degradation [58,59,60], ubiquilins have been implicated in retinal health and disease [61,62,63,64] with pathologies that include retinitis pigmentosa, macular degenerations, glaucoma, diabetic retinopathy, and age-related impairments [63,65,66,67]. On the other hand, increased proteasomal activity has been found to support, photoreceptor survival in inherited retinal degeneration [68], which provided a further rationale to consider UBQLN1 upregulation by E2 as a marker in the context of neuroprotective effects.

ATP5F1B was another protein found in an IPA^®^ network associated in part with ER signaling (Figure S8), justifying its inclusion among the estrogen-regulated markers for assessment by targeted proteomics [69] in the male rat retina. This was further augmented by the recognition that high energy demands in the inner retina were tightly coupled with the functional activity of its neurons [70,71]. Moreover, downregulation of ATP synthase has been identified as an early response to oxidative stress in the retina indicating damage of the tissue [72]. Overall, mitochondrial dysfunction has been implicated in the pathophysiology of prevalent retinal diseases such as diabetic retinopathy, age-related macular degeneration, and glaucoma [73,74,75]. Therefore, mitochondria have been promising therapeutic targets in the retina [76,77,78]. 

As these proteomics data fully supported the beneficial effects of E2 in the male rat retina, we then investigated whether targeted E2 delivery into the male retina can also be achieved by DHED eye drops. Of note, both E2 and DHED eye drops were formulated in saline containing HPβCD. Cyclodextrins are well-known excipients, allowing for aqueous formulation of poorly water-soluble compounds, such as E2 [10,11]. They also enhance corneal permeability to decrease systemic drug absorption, increase stability, and decrease local irritation [79,80]. 

As such, we investigated whether the site-specific prodrug metabolism seen in female animals [13] would also occur in the male retina, as gender has been implicated as a factor in several aspects of the CNS [27,28,29]. While therapeutic safety is mission-critical in the context of potential estrogen-based neurotherapies [26], it is especially important for males to avoid peripheral exposure to the hormone. Previously, we have shown that DHED, the bioprecursor prodrug of E2 (Figure 1), metabolized in the female retina to E2 without peripheral exposure to the hormone [11]. In the current study, we have shown that this metabolism takes place in the male retina as well (Table 4, Figure 5) with concomitant target engagements characteristic to E2 (Figure 6). Gender is apparently not a potent modifier of the DHED-to-E2 enzymatic conversion in the retina that is an extension of the CNS [81]. Importantly, in male rats and rabbits we have also shown that our non-invasive topical DHED treatment did not increase circulating E2 levels (Table 4 and Figure 5), implying therapeutic safety (Figure 4). Considered altogether, E2’s beneficial effects can be specifically and selectively retained within the retina in a gender-independent fashion using DHED eye drops. The eye drops are fully expected to reach the retina predominantly via the transcorneal route [11,82], owing to DHED’s favorable physicochemical properties in this context [30]. Therefore, the investigation presented here provides further rationale to explore the full potential of our retina-targeted E2 delivery by the DHED prodrug approach for a safe and efficacious neuroprotection, regardless of gender.

## Figures and Tables

**Table 1:** (**A**) Molecular and cellular functions, as well as (**B**) physiological system development and function represented by significantly affected retina proteins identified by label-free quantitative proteomics in ORX Brown Norway rats having received E2 eye drops (q.d., 3 weeks).

(A)		
Represented Process	Number of Associated Molecules	*p*-Value of Overlap
Cellular function and maintenance	42	7.01·10^−3^–5.59·10^−6^
Protein synthesis	30	7.35·10^−3^–5.63·10^−5^
Cellular assembly and organization	39	7.01·10^−3^–1.34·10^−5^
Cell death and survival	55	7.01·10^−3^–1.58·10^−5^
Protein degradation	15	3.93·10^−3^–2.18·10^−5^
**(B)**		
**Associated Physiology**	**Number of Linked Molecules**	***p*** **-Value of Overlap**
Visual system development and function	14	5.74·10^−3^–1.83·10^−6^
Embryonic development	19	7.01·10^−3^–1.91·10^−5^
Nervous system development and function	32	7.50·10^−3^–1.91·10^−5^
Organ development	18	7.01·10^−3^–1.91·10^−5^

**Table 2:** Canonical pathways and their molecular targets in the network shown in Figure 2.

Canonical Pathway	Molecular Targets	Z-Score ^1^	*P*
Protein ubiquitination	20S proteasome; 26S proteasome; CRYAA/CRYAA2; CRYAB; Hsp70; HSP; HSPH1; immunoproteasome Pa28/20S, proteasomePA700/20S, PSMAE, PSMA4, PSMB3, ubiquitin	N/A	1.94·10^−4^
Synaptogenesis signaling	Akt; HSP70; HSP	2	3.42·10^−4^

^1^ Positive value: activation of the canonical pathway; N/A: no prediction can be made.

**Table 3:** Canonical pathways and their molecular targets in the network shown in Figure 3 ^1^.

Canonical Pathway	Molecular Targets	*p*
Protein ubiquitination	CRYAA/CRYAA2, DNAJC28, HSPA13, HSPB2, Ubiquitin	4.66·10^−2^
GABA receptor signaling	Ubiquitin, UBQLN1	2.24·10^−2^
Mitochondrial dysfunction	ATP5F1B, GLRX2, ATP5MC1, CASP9	2.90·10^−2^

^1^ No Z-score prediction was made by IPA^®^ for activation or suppression.

**Table 4:** Topical DHED does not expose the periphery to E2 but produces a significant E2 level in the male rat retina after three-week q.d. eye drops (10 μL, 0.1% *w/v* in 20% *w/v* HPβCD in saline solution). Tissues were collected 24 h after the last treatment. Control animals received vehicle eye drops only. E2 level measured 24 h after a single prodrug eye drop served as a reference. Data are given as average ± SD, *n* = 10 for serum and *n* = 5 for retina. * Statistically significant difference from vehicle treatment. ** Statistically significant difference from DHED treatment (ANOVA followed by Tukey test, *p* < 0.05).

Treatment (Eye drop, q.d.)	E2 in Retina (pg/g)	E2 in Serum (pg/mL)
Vehicle	130 ± 41	3.3 ± 1.5
DHED, 3-week, q.d.	482 ± 77 *	4.5 ± 2.1
DHED, single dose	397 ± 105 *	4.2 ± 1.8
d_3_-DHED, single dose	413 ± 130 * (d_3_-E2) & 125 ± 18 (E2)	N.D. ^1^ (d_3_-E2) & 4.3 ± 2.1 (E2)
E2, 3-week, q.d.	569 ± 169 *^,^**	414 ± 79 *^,^**

^1^ N.D. denotes not detected.

## Data Availability

The mass spectrometry proteomics data have been deposited to the ProteomeXchange Consortium via the PRIDE [83] partner repository with the dataset identifier PXD027902 and 10.6019/PXD027902.

## Supplementary Materials

The following are available online at https://www.mdpi.com/article/10.3390/pharmaceutics13091392/s1. Table S1: (**a**) List of identified and validated proteins (Scaffold 4.9.0: >95% and >99.9% probability by Peptide Prophet and protein Prophet, respectively, with minimum of two unique peptides) in the retina of control and E2-treated ORX males, along with their gene ontology (GO); (**b**) List of all identified peptides validated by Scaffold and used for quantitation by spectral counting and selection of proteotypic peptides for PRM-based targeted proteomics, Table S2: A list of E2-regulated retinal proteins in ORX male rats, Table S3: PRM setup for targeted proteomics to quantify fold changes of CRYAA, CRYBB, UBQLN1, and ATP5F1B with isotope labeled internal standards using Skyline, Table S4: Peptide transition list results from Skyline used for targeted quantification by PRM, Figure S1: IPA^®^ network linked to development disorder, hereditary disorder, organismal injury, and abnormalities, Figure S2: IPA^®^ network linked to cellular assembly and organization, cellular function and maintenance, protein synthesis, Figure S3: IPA^®^ network linked to cancer, hematological disease, immunological disease, Figure S4: IPA^®^ network linked to cellular development, cellular growth and proliferation, hematological system development and function, Figure S5: IPA^®^ network linked to cancer, cell death and survival, cell signaling, Figure S6: IPA^®^ network linked to cancer, organismal injury and abnormalities, Figure S7: IPA^®^ network linked to cell cycle, cellular development, connective tissue development and function, Figure S8: IPA^®^ network linked to cancer, molecular transport, organismal injury and abnormalities, Figure S9: Overlapping IPA^®^ networks.
