# Supplementary material for: Proteomics-Based Retinal Target Engagement Analysis and Retina-Targeted Delivery of 17β-Estradiol by the DHED Prodrug for Ocular Neurotherapy in Males
Source: Pharmaceutics. 2021 Sep 2;13(9):1392. doi: 10.3390/pharmaceutics13091392 (PMC8466286; doi:10.3390/pharmaceutics13091392)
Supplement: Supplementary file 1 [file pharmaceutics-13-01392-s001.zip › pharmaceutics-1321752_SupplementaryFiguresS1-S9.pdf]

(**a**)

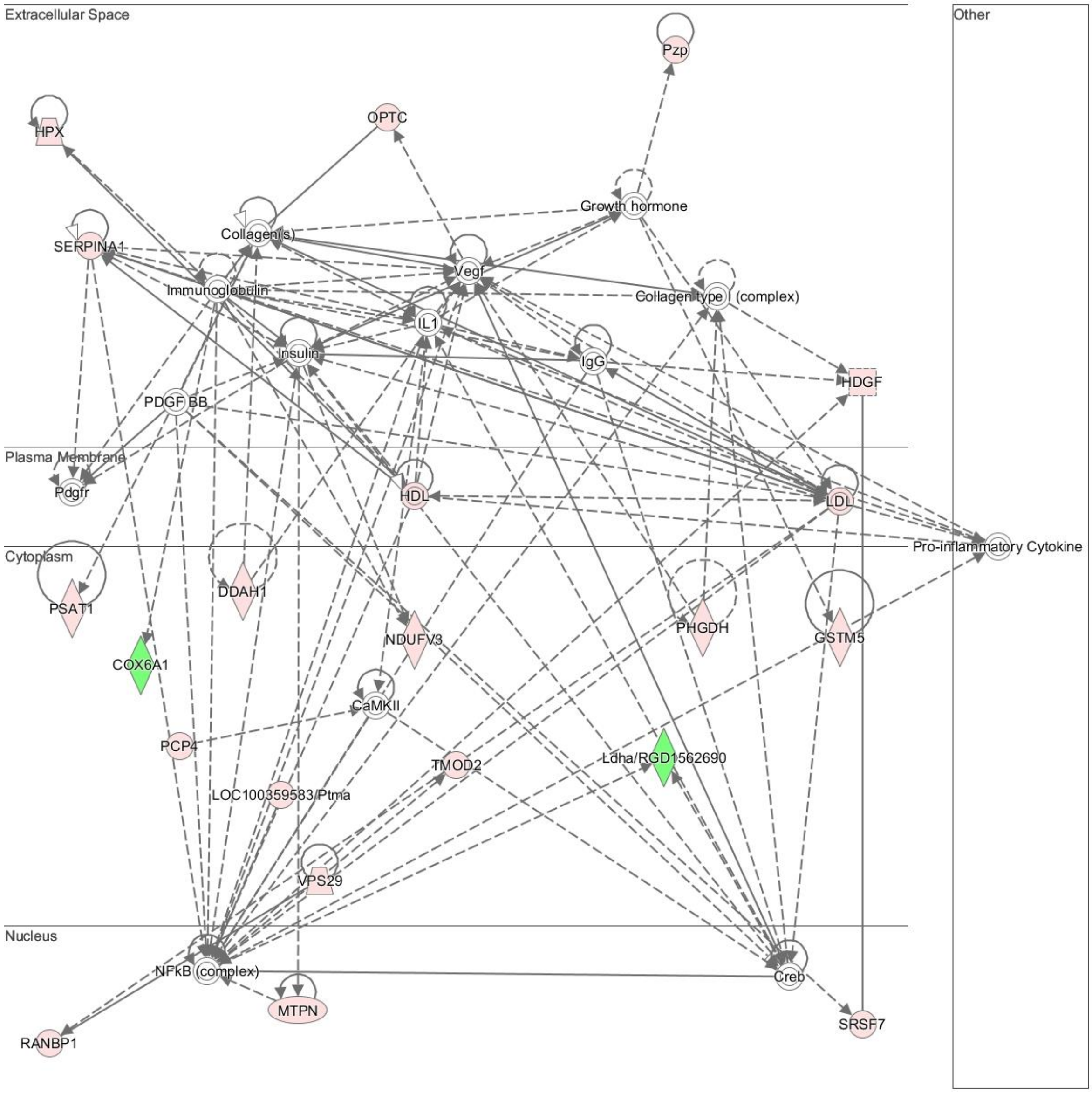

(**b**)

Canonical pathway	Target molecules	Z-score	*P*-value
Clathrin-mediated endocytosis signaling	HDL, Insulin, LDL, PDGF BB, SERPOMA1, Vegf	N/A	9.99E-02
Iron homeostasis signaling	Creb, HDL, HPX, PDGF BB, PDGFR, Pro-inflammatory cytokine	N/A	8.51E-03

Figure S1. IPA® network linked to development disorder, hereditary disorder, organismal injury and abnormalities (**a**). The network was assembled from E_2-regulated proteins in the retina of castrated male rats through identification by label-free shotgun proteomics. Red symbols: upregulated, green symbols: downregulated by the hormone; solid line: direct relationship, dashed line: indirect relationship. For further details on symbols and relationships, see http://qiagen.force.com/KnowledgeBase/articles/Basic_Technical_Q_A/Legend. Inset table (**b**): Top canonical pathways and their corresponding molecular targets associated with the network shown. Z-scores indicate activation or suppression of the corresponding pathway (positive or negative values, respectively; N/A: no prediction can be made).

(**a**)

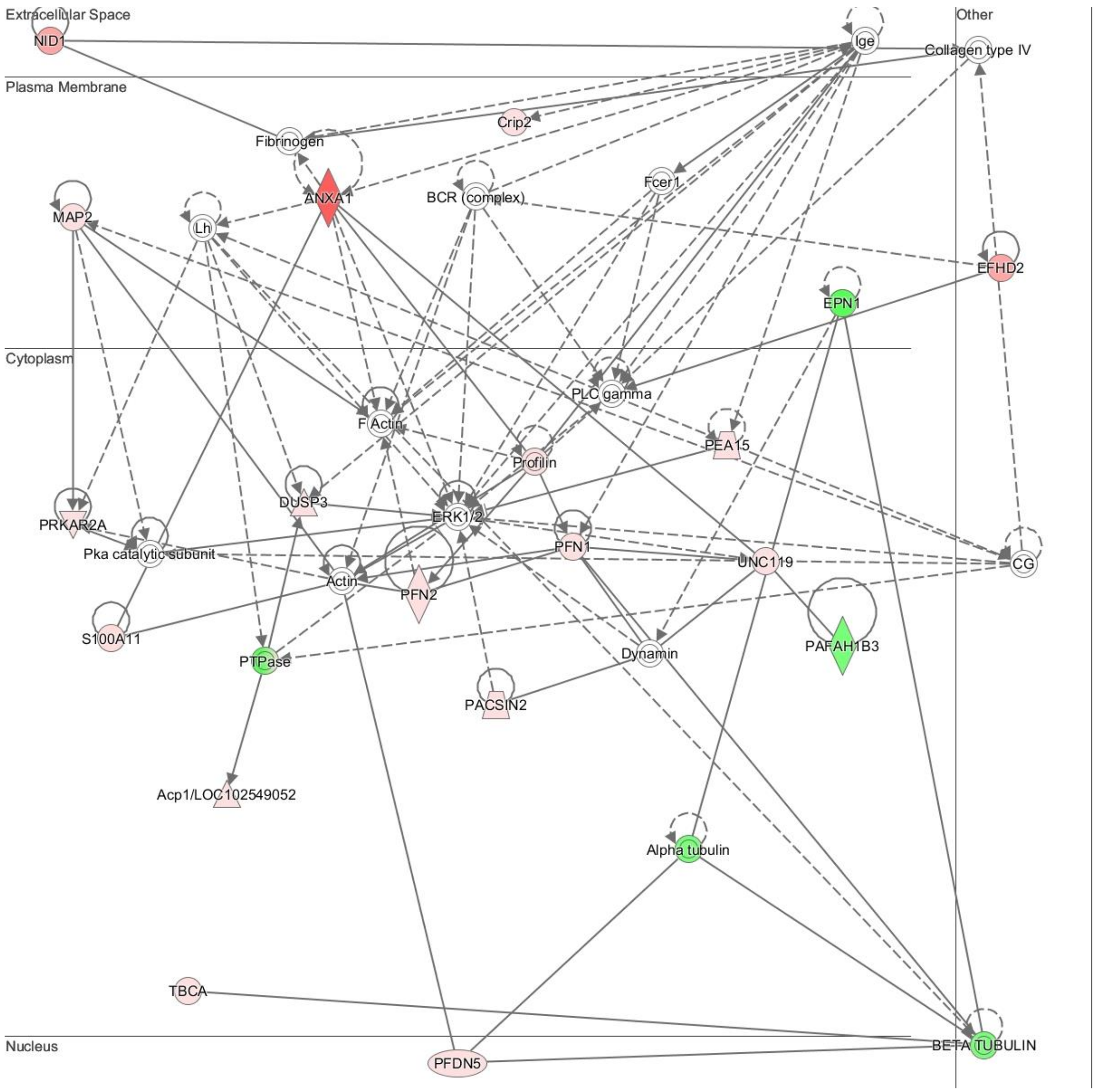

(**b**)

**Canonical pathway**	**Target molecules**	**Z-score**	***P*-value**
Axonal guidance signaling	A tubulin, beta tubulin, ERK1/2, PFN1, PFN2, Pka catalytic subunit, PLC gamma, PRKAR2A, Profilin, PTPase	N/A	2.70E-02
Protein kinase A signaling	Acp1/loc102549052, DUSP3, ERK1/2, Pka catalytic subunit, PLC gamma, PRKAR2A, PTPase	0.45	8.23E-02

Figure S2. IPA® network linked to cellular assembly and organization, cellular function and maintenance, protein synthesis (**a**). The network was assembled from E_2-regulated proteins in the retina of castrated male rats through identification by label-free shotgun proteomics. Red symbols: upregulated, green symbols: downregulated by the hormone; solid line: direct relationship, dashed line: indirect relationship. See http://qiagen.force.com/KnowledgeBase/articles/Basic_Technical_Q_A/Legend for further details on symbols and relationships. Inset table (**b**): Top canonical pathways and their corresponding molecular targets associated with the network shown. Z-scores indicate activation or suppression of the corresponding pathway (positive or negative values, respectively; N/A: no prediction can be made).

(**a**)

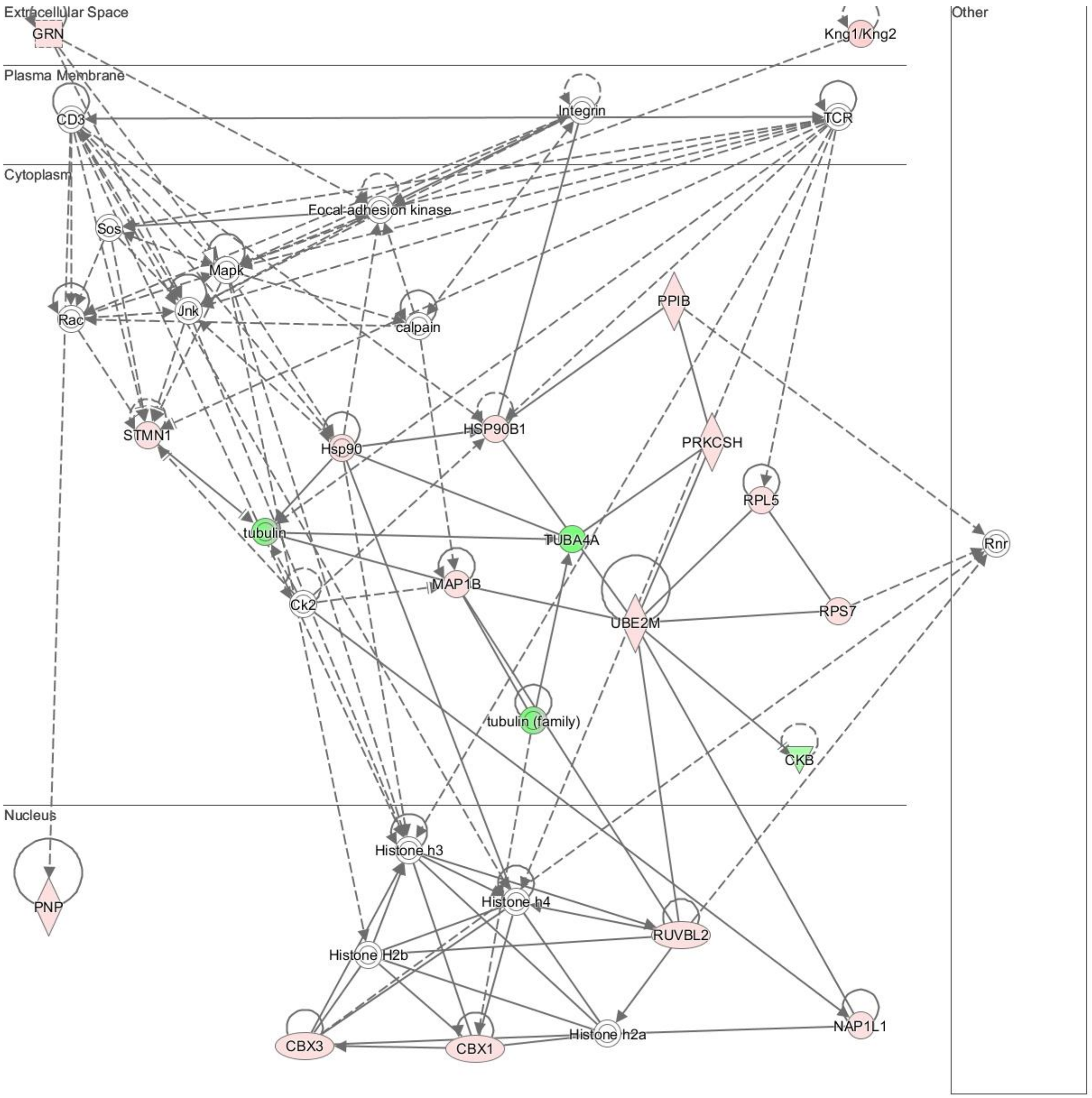

(**b**)

**Canonical pathway**	**Target molecules**	**Z-score**	***P*-value**
Sirtuin signaling	Histone h3, Histone h4, JNK, Mapk, TUBA4A, tubulin, tubulin family	N/A	2.68E-02
Axonal guidance signaling	Focal adheshion kinase, integrin, Mapk, Rac, Sos, TUBA4A, Tubulin, tubulin family	N/A	2.70E-02

Figure S3. IPA® network linked to Cancer, hematological disease, immunological disease (**a**). The network was assembled from E_2-regulated proteins in the retina of castrated male rats through identification by label-free shotgun proteomics. Red symbols: upregulated, green symbols: downregulated by the hormone; solid line: direct relationship, dashed line: indirect relationship. For further details on symbols and relationships, see http://qiagen.force.com/KnowledgeBase/articles/Basic_Technical_Q_A/Legend. Inset table (**b**): Top canonical pathways and their corresponding molecular targets associated with the network shown. Z-scores indicate activation or suppression of the corresponding pathway (positive or negative values, respectively; N/A: no prediction can be made).

(a)

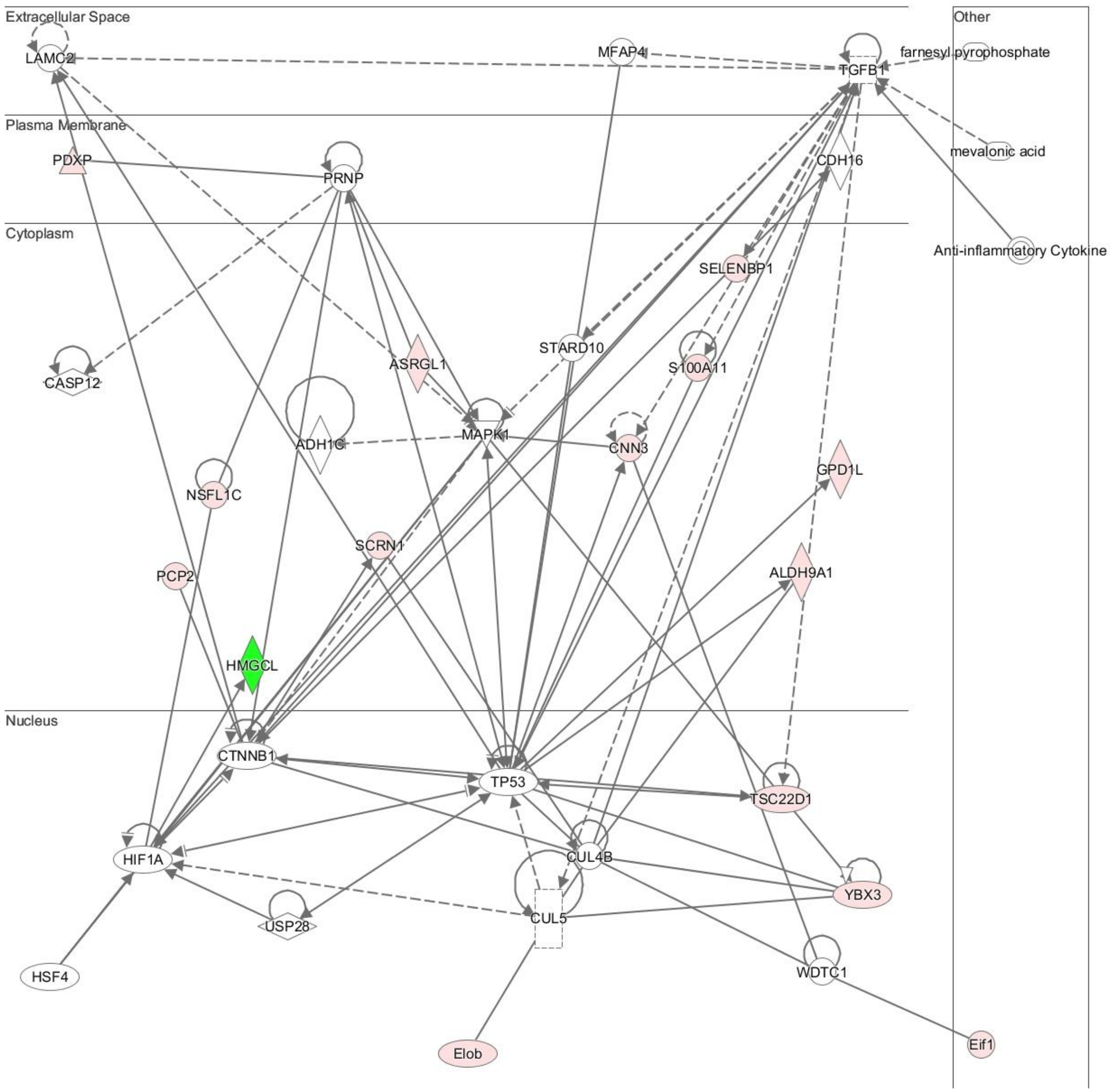

(b)

Canonical pathway	Target molecules	Z-score	*P*-value
Coronavirus pathogenesis pathway	Anti-inflammatory cytokine, HIFIA, MAPK1, TGFB1, TP53	N/A	2.76E-02
Sirtuin signaling	HIFIA, MAPK, TP53	N/A	2.68E-02

Figure S4. IPA® network linked to cellular development, cellular growth and proliferation, hematological system development and function (**a**). The network was assembled from E_2-regulated proteins in the retina of castrated male rats through identification by label-free shotgun proteomics. Red symbols: upregulated, green symbols: downregulated by the hormone; solid line: direct relationship, dashed line: indirect relationship. See http://qiagen.force.com/KnowledgeBase/articles/Basic_Technical_Q_A/Legend for further details on symbols and relationships. Inset table (**b**): Top canonical pathways and their corresponding molecular targets associated with the network shown. Z-scores indicate activation or suppression of the corresponding pathway (positive or negative values, respectively; N/A: no prediction can be made).

(**a**)

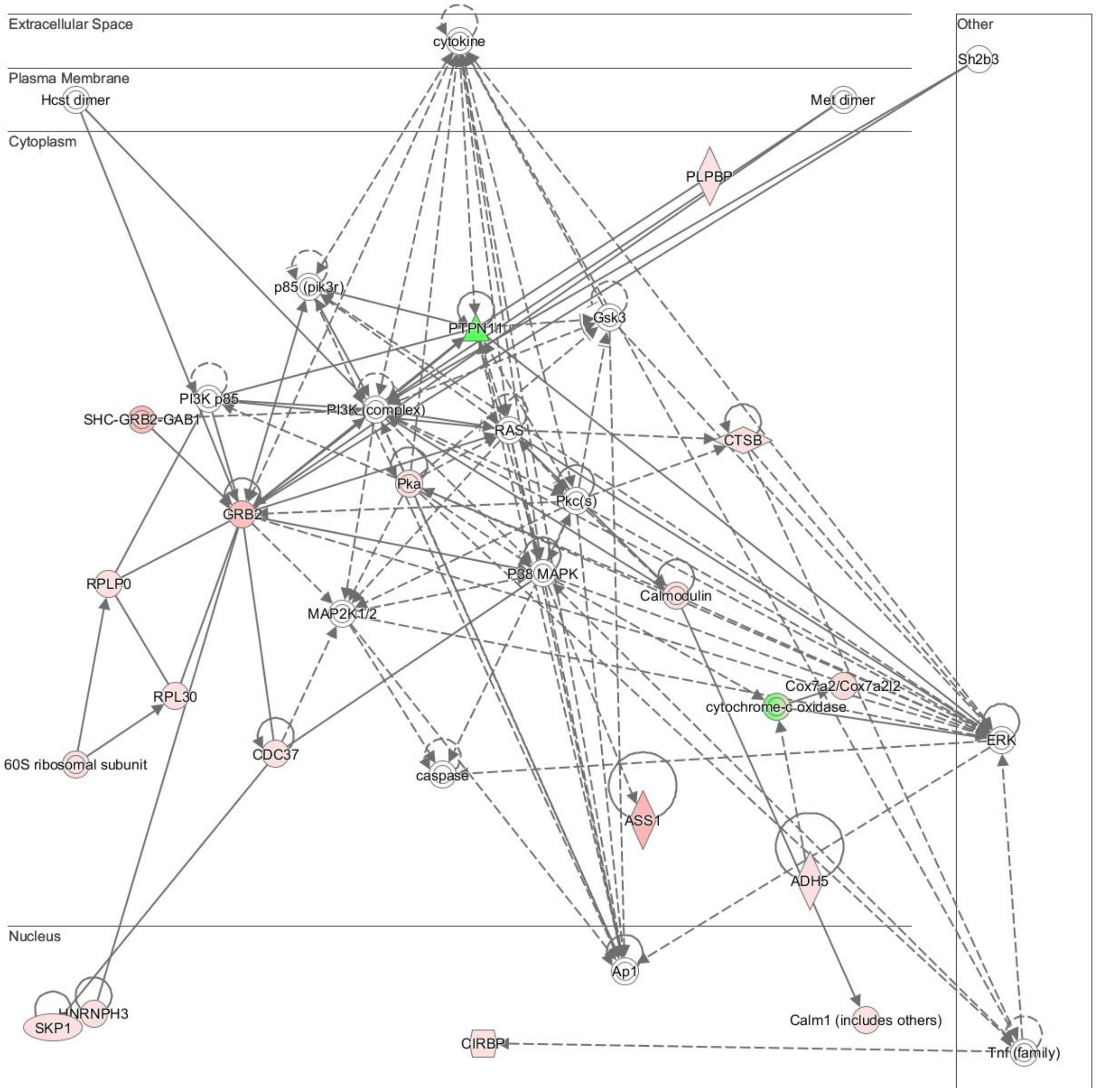

(**b**)

**Canonical pathway**	**Target molecules**	**Z-score**	***P*-value**
Synaptogenesis signaling pathway	Calm1, calmodulin, EKR, GRB2, Gsk3, P38 MAPK, P85, PI3K complex, PI3K p85, Pka, Pkc's, RAS, SHC-GRB2-GAB1	2	3.42E-02
Estrogen receptor signaling	AP1, EKR, GRB2, Gsk3, MAP2K1/2, P38 MAPK, P85, PI3K complex, PI3K p85, Pka, Pkc, RAS, SHC-GRB2-GAB1	N/A	8.37E-02

Figure S5. IPA® network linked to cancer, cell death and survival, cell signaling (**a**). The network was assembled from E_2-regulated proteins in the retina of castrated male rats through identification by label-free shotgun proteomics . Red symbols: upregulated, green symbols: downregulated by the hormone; solid line: direct relationship, dashed line: indirect relationship. For further details on symbols and relationships, see http://qiagen.force.com/KnowledgeBase/articles/Basic_Technical_Q_A/Legend. Inset table (**b**): Top canonical pathways and their corresponding molecular targets associated with the network shown. Z-scores indicate activation or suppression of the corresponding pathway (positive or negative values, respectively; N/A: no prediction can be made).

(**a**)

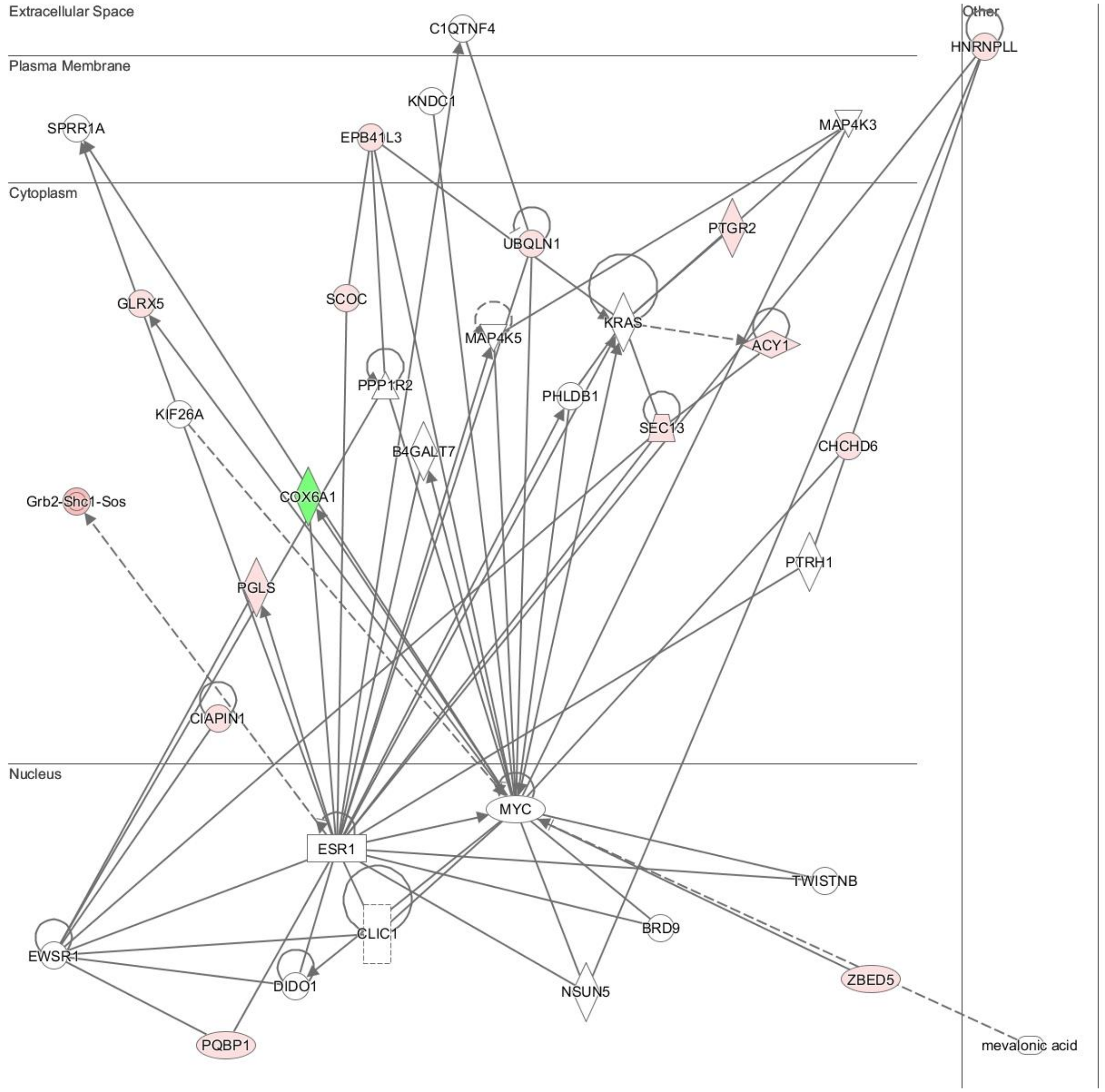

(**b**)

**Canonical pathway**	**Target molecules**	**Z-score**	***P*-value**
EIF2 signaling	Grb2-shc1-sos, KRAS, MYC	2	9.56E-03
Estrogen receptor signaling	ESR1, Grb2-shc1-sos, KRAS, MYC	N/A	8.37E-02

Figure S6. IPA® network linked to cancer, organismal injury and abnormalities (**a**). The network was assembled from E_2-regulated proteins in the retina of castrated male rats through identification by label-free shotgun proteomics. Red symbols: upregulated, green symbols: downregulated by the hormone; solid line: direct relationship, dashed line: indirect relationship. For further details on symbols and relationships, see http://qiagen.force.com/KnowledgeBase/articles/Basic_Technical_Q_A/Legend. Inset table (**b**): Top canonical pathways and their corresponding molecular targets associated with the network shown. Z-scores indicate activation or suppression of the corresponding pathway (positive or negative values, respectively; N/A: no prediction can be made).

(a)

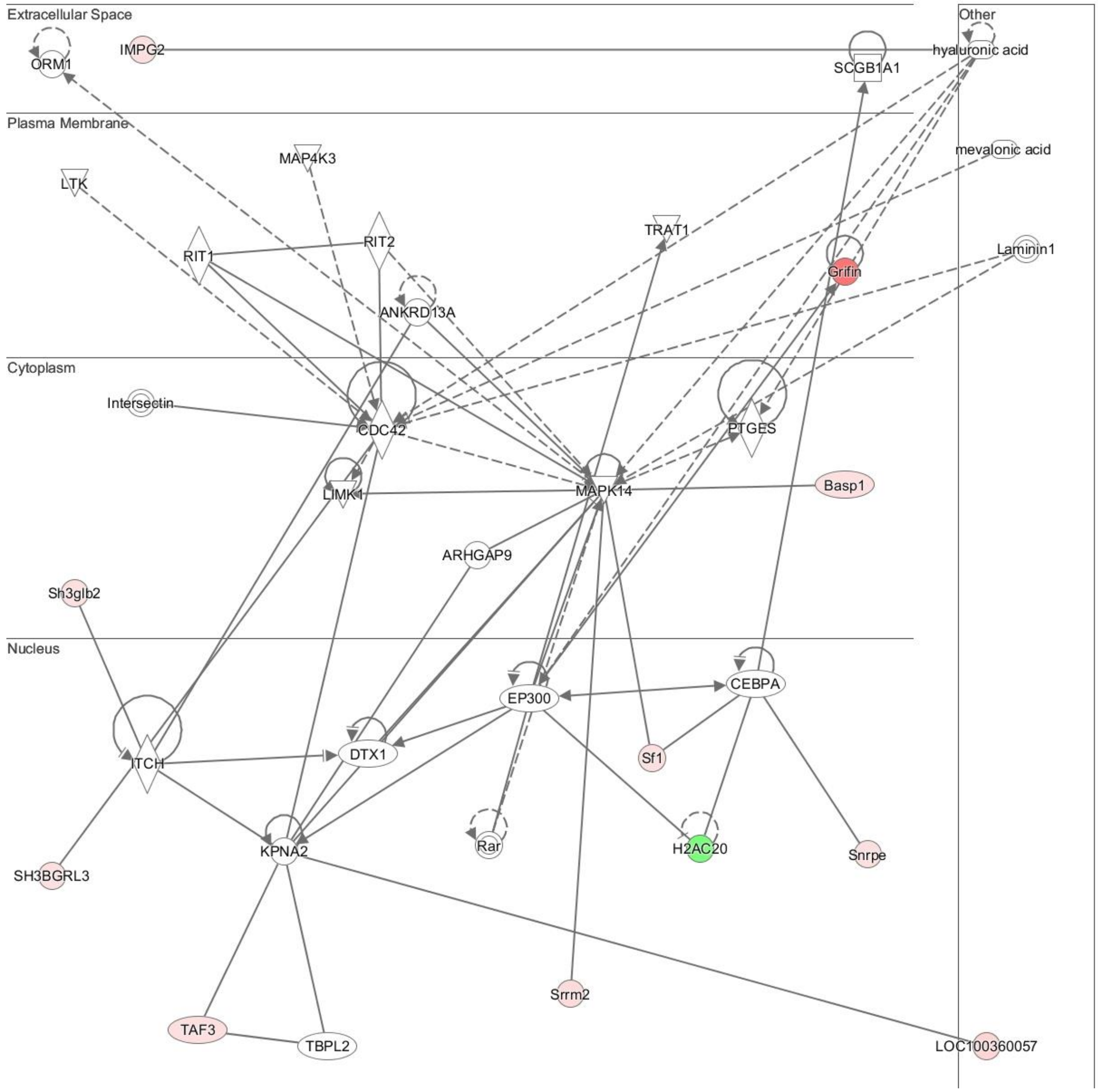

(b)

Canonical pathway	Target molecules	Z-score	*P*-value
Synaptogenesis signaling pathway	CDC42, Intersectin, LIMK1, MAPK14	2	3.42E-02
Axonal guidance signaling	CDC42, Intersectin, LIMK1	N/A	2.70E-02

Figure S7. IPA® network linked to cell cycle, cellular development, connective tissue development and function (**a**). The network was assembled from E_2-regulated proteins in the retina of castrated male rats through identification by label-free shotgun proteomics. Red symbols: upregulated, green symbols: downregulated by the hormone; solid line: direct relationship, dashed line: indirect relationship. For further details on symbols and relationships, see http://qiagen.force.com/KnowledgeBase/articles/Basic_Technical_Q_A/Legend. Inset table (**b**): Top canonical pathways and their corresponding molecular targets associated with the network shown. Z-scores indicate activation or suppression of the corresponding pathway (positive or negative values, respectively; N/A: no prediction can be made).

(a)

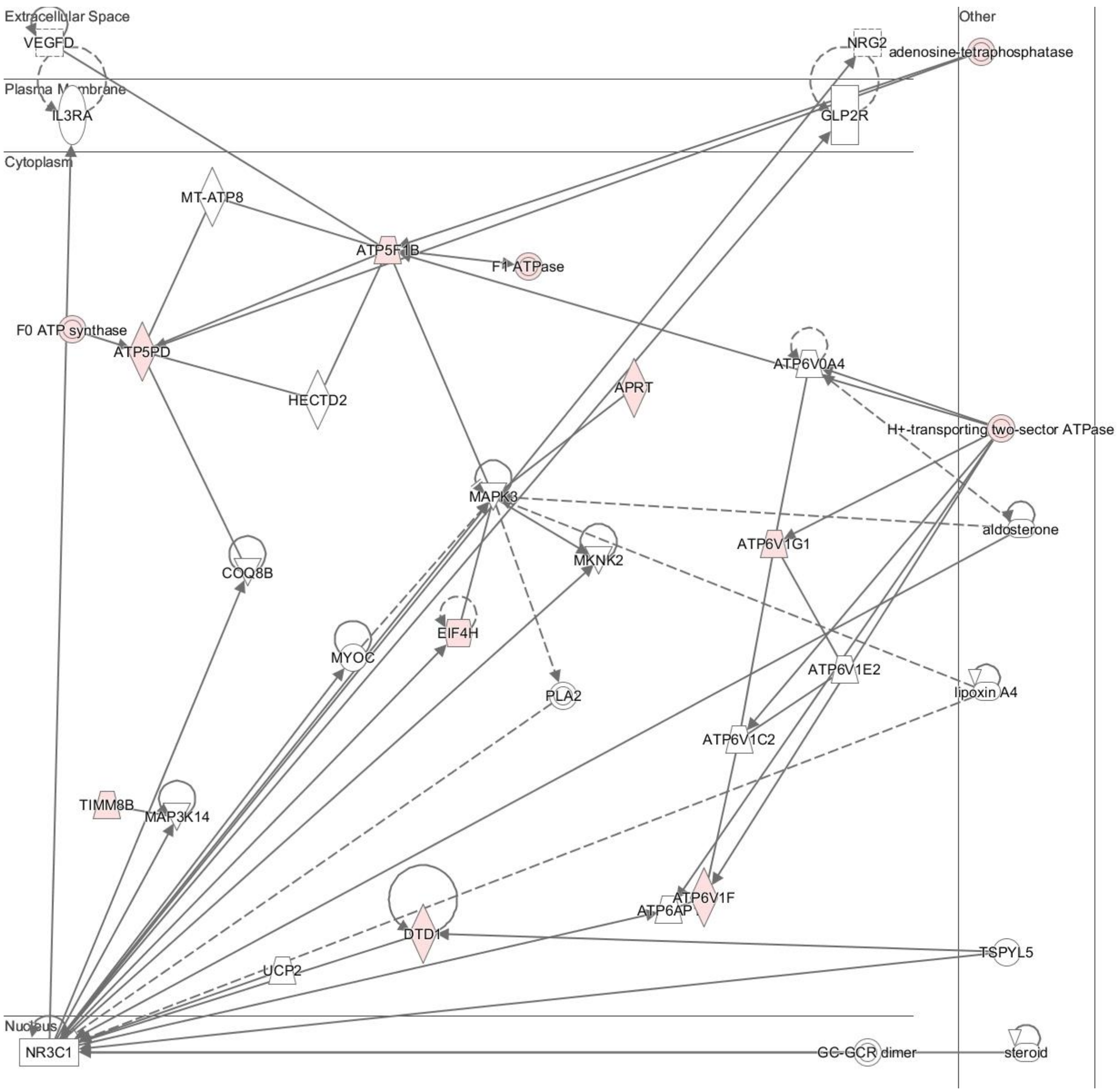

(b)

**Canonical pathway**	**Target molecules**	**Z-score**	***P*-value**
Estrogen receptor signaling	Adenosine tetraphosphatase, aldosterone, ATP5F1B, FO ATP synthase, GC-GCR dimer, H+ transporting two sector ATPase, IL3RA, MAP3K14, MAPK3, NR3C1, PLA2	N/A	8.37E-02
Oxidative phosphorylation	Adenosine tetraphosphatase, aldosterone, ATP5F1B, FO ATP synthase, GC-GCR dimer, H+ transporting two sector ATPase	1.34	4.19E-04

Figure S8. IPA® network linked to cancer, molecular transport, organismal injury and abnormalities (**a**). The network was assembled from E_2-regulated proteins in the retina of castrated male rats through identification by label-free shotgun proteomics. Red symbols: upregulated, green symbols: downregulated by the hormone; solid line: direct relationship, dashed line: indirect relationship. For further details on symbols and relationships, see http://qiagen.force.com/KnowledgeBase/articles/Basic_Technical_Q_A/Legend. Inset table (**b**): Top canonical pathways and their corresponding molecular targets associated with the network shown. Z-scores indicate activation or suppression of the corresponding pathway (positive or negative values, respectively; N/A: no prediction can be made).

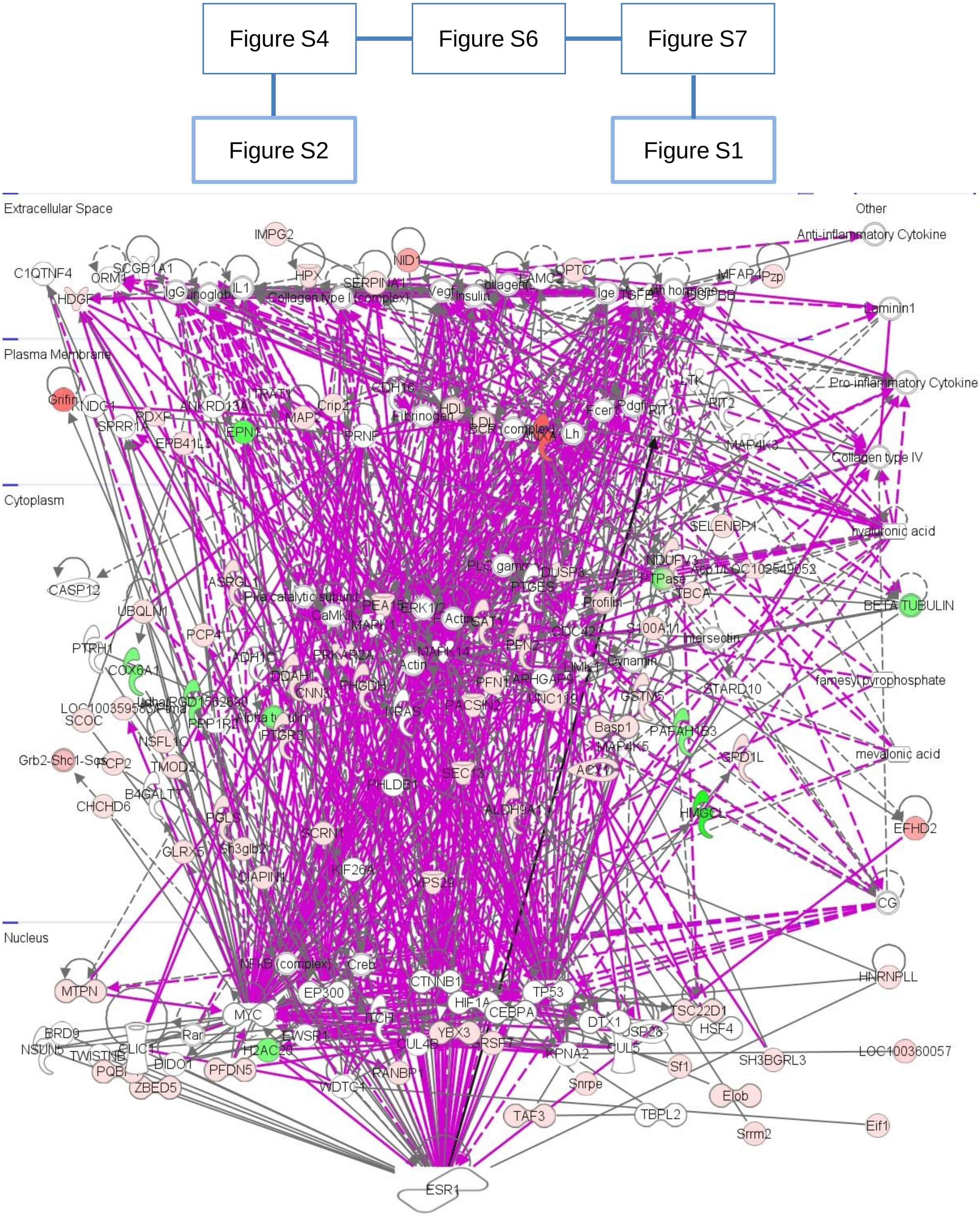

Figure S9. Overlapping IPA® networks.
